# Expectations vs. actual behavior of a social robot: An experimental investigation of the effects of a social robot’s interaction skill level and its expected future role on people’s evaluations

**DOI:** 10.1371/journal.pone.0238133

**Published:** 2020-08-21

**Authors:** Aike C. Horstmann, Nicole C. Krämer

**Affiliations:** Social Psychology: Media and Communication, University of Duisburg-Essen, Duisburg, Germany; Nanyang Technological University, SINGAPORE

## Abstract

Since social robots are increasingly entering areas of people’s personal lives, it is crucial to examine what affects people’s perceptions and evaluations of these robots. In this study, three potential influences are examined: 1) the robot’s level of interaction skills, 2) the robot’s expected future role as a helpful assistant or a threatening competitor, and 3) people’s individual background with regard to robots and technology in general. In an experimental lab study with a 2x2 between-subjects-design (N = 162), people read a vignette describing the social robot Nao either as assistant or competitor and subsequently interacted with Nao, which either displayed high or low interaction skills. Results of a structural equation model show that the robot’s interaction skill level had the strongest effect, with a low level leading to a negative evaluation of the robot’s sociability and competence and subsequently a negative general evaluation of the interaction with the robot. A robot which was expected to become a competitor was also evaluated as less sociable than a robot expected to become an assistant. Overall, in case of an actual interaction with a social robot, the robot’s behavior is more decisive for people’s evaluations of it than their expectations or individual backgrounds.

## Introduction

Since social robots are taking over roles which are traditionally filled by humans and thus are becoming a growing part of our society, it is crucial to examine what affects people’s evaluation and acceptance of these robots. How a robot is evaluated depends on several factors like the robot’s appearance [1–4], its nonverbal behavior [3, 5], other behavioral aspects (e.g., predictability: [6, 7] and cooperativeness: [8]) and media portrayals [9–12]. This study focuses on three aspects: the robot’s behavior, the user’s expectation, and the user’s individual background.

With regard to behavior, the robot’s level of interaction skills should affect people’s evaluations tremendously for several reasons. First of all, behavior in general appears to play a major role in interactions with non-human entities [13]. Furthermore, interaction skills are essential for a social robot to fulfill its function [14] and according to Davis [15], a technology’s perceived usefulness is linked to people’s attitude towards and usage intentions of this technology. When a robot displays low interaction skills, this robot and the interaction with it should be evaluated differently compared to when the robot displays high interaction skills.

With regard to expectations, most people have not personally interacted with a social robot yet and thus have to draw information from other sources, which are often mass media reports [11]. Media representations of “good” as well as “bad” fictional robot characters form people’s attitude towards and expectations about social robots and lead to double-minded feelings [10]. Since expectations serve as perceptual filters of the reality [16, 17], whether people expect a social robot to become a helpful assistant or a threatening competitor should affect how people evaluate the robot and the interaction with it. Moreover, when the robot is expected to become a competitor, high interaction skills should be perceived more threatening and thus more negatively than low skills.

A preliminary analysis of a small part of the data already indicated that the robot’s level of interaction skills as well as people’s expectation regarding the robot’s future role both have an impact on people’s evaluation of this robot as well as on the evaluation of the interaction with the robot t [18]. However, when examining human-robot interaction processes, people’s individual background needs be taken into account as well. In this work, we additionally look at people’s technical affinity [19], their locus of control when using technologies [20], their previous experiences with real or fictional robots and their negative expectancies regarding robots. To get a more comprehensive picture, the focus of this work is to look at these different influences (behavior, expectation, and individual background) in one structural equation model to be able to examine how they relate to each other while affecting how a robot and consequently the interaction with it are evaluated. Summing up, this study examines the main and interaction effects of a social robot’s interaction skill level, the robot’s expected future role and users’ personality variables to find out which aspects play what kind of role in people’s perceptions and evaluations of a robot with which they just interacted.

### A social robot’s level of interaction skills

How a social robot behaves while interacting with people, should play a central role for people’s evaluation of this robot. One reason for this assumption is that behavior in general was found to have a tremendous impact in interactions with non-human entities [13]. According to Rickenberg and Reeves [13], the evaluation of a character “depends on what the character does, what it says, and how it presents itself” (p. 55). Against this background, this study looks at the effects of different behaviors of a social robot, particularly whether this robot displays poor or sophisticated social interaction skills.

Since the media’s focus is often predominantly on the success and progress of a technology and neglects problems and setbacks, this leads people to form exorbitantly high expectations about social robots and their skills [11]. In this vein, robots are generally expected to be performance-oriented, i.e., efficient, reliable, precise, rational, and perfectionist [21–23]. Likewise, research by Kwon et al. [24] showed that people tend to generalize social capabilities for humanoid robots, which also results in very high expectations for these robots. Looking at social robots specifically, people expect them to be able to talk to them, to understand them and to react to them in a sophisticated manner [11]. One of the main characteristics of socially interactive robots is the ability to communicate with high-level dialogue [25]. Even though there are several definitions of social robots, the ability to interact with its environment to some degree (or at least to give the impression to do so) resembles the core definition of a social robot [14]. Without adequate interaction skills, social robots would not be able to fulfill their purpose of interacting with people in a social way and, thus, would not be very useful in their common areas of application like home companionship, nursing care, entertainment, and office/hotel assistance [14]. According to the findings obtained while developing the well-established Technology Acceptance Model, perceived usefulness has a powerful effect on the attitude towards using as well as on the actual use of a technology [15]. If a social robot has low interaction skills, this should reduce its perceived usefulness and consequently lead to detrimental consequences for the evaluation of this robot. Research showed that when a robot’s functions fall behind people’s expectations this leads to negative communication outcomes like disappointment, mistrust, and rejection [26]. Thus, a robot displaying low levels of interaction skills should result in a negative evaluation of the robot’s sociability and competence. How the robot is evaluated should then also affect the general evaluation of the interaction with the robot. Against this background, the following hypotheses were postulated:

**H1**: A robot with a low level of interaction skills leads to a more negative evaluation of a) the robot’s sociability and b) its competence, which consequently leads to c) a more negative general evaluation of the interaction with the robot, compared to a robot with a high interaction skill level.

### A social robot’s expected future role

In general, there are mainly two prominent prospects for social robots–one which is feared and one which is desired. Since both images are promoted in media, this leads people to have double-minded feelings towards robots [10].

On the one hand, there is this negative view on social robots becoming competitors. People are worried about autonomous robots and loss of control, which is often accompanied by a fear that humans will be either replaced or dominated by robots (known as “Frankenstein Syndrom”; [27–29]). Mass media, particularly science fiction formats, have a great influence on people’s image of social robots and promote the idea of robots developing their own agenda and revolting against humans [30]. In this vein, a study by Horstmann and Krämer [11] showed that the more “bad” fictional robot characters people know, the more do they fear robots to become superior and a threat to humans. To extend these survey-based findings, this study aims to examine in a systematic-experimental way whether a negative expectation about a robot affects how people evaluate this robot after they interacted with it. It is assumed that interacting with a robot which is expected to learn from humans to become better and more efficient than them and to take away tasks from them is undesirable and that this robot as well as the interaction with it are evaluated poorly.

On the other hand, there is also this positive image of social robots functioning as assistants, either in domestic, public, or work environments [11]. This idea of having an electronic help, which makes life easier by carrying out tasks that are unpleasant or strenuous, is very appealing to most people [11, 28, 31]. Thus, people should evaluate a robot which is expected to assist humans with various tasks in the future positively and should also enjoy the interaction with this robot.

In order to examine whether manipulating people’s expectations by framing the robot in a negative light (by describing it as competitor working against humans) or in a positive light (by describing it as assistant working for humans) influences how they evaluate the robot and consequently the interaction with it, the following hypotheses were formulated:

**H2**: When a robot is expected to take over the role of a competitor, this leads to a more negative evaluation of a) the robot’s sociability and b) its competence, which consequently leads to c) a more negative general evaluation of the interaction with the robot, compared to when the robot is expected to become an assistant.

### A social robot’s expected future role influencing the perception of its interaction skill level

It is crucial for the acceptance and successful employment of social robots to find out which influencing variables may interact with each other while affecting the evaluation of a robot. It is known from interpersonal studies, that how a person’s behavior is interpreted is heavily influenced by how desirable or rewarding it is to interact with the person [16]. It is a function of all pre-interactional relationship and communicator characteristics (e.g., personality, reputation, and nature of the relationship) and all interactional behaviors (e.g., an amusing communication style or positive feedback; [32]). Transferring this to human-robot interaction, how a robot’s behavior is perceived should also be affected by how desirable it is for people to interact with this robot. Since there are ambiguous views on social robots, whether the robot is expected to become a threatening competitor or a helpful assistant in the future should affect how the robot’s level of interaction skills are perceived.

More specifically, a social robot’s skills might be perceived differently depending on how people think this robot will use these skills in the future. In case of a robot which is expected to become a competitor working against humans, high interaction skills could be perceived as daunting. In science fiction, robots are often portrayed as highly skilled and extremely intelligent, often surpassing humans and threatening to obtain world domination [27, 33–35]. Research has shown that these are scenarios that people are also afraid of in real life [28, 29], especially when they are able to recall a lot of these negative fictional robot characters [11]. Thus, in case of a threatening competitor robot, people would most likely prefer this robot to have low interaction skills. However, if a robot is expected to be a helpful assistant working for humans, people would probably like to have this robot to be equipped with sophisticated interaction skills. Against this background, this study aims to examine how different levels of a social robot’s interaction skills affect people’s evaluation of the robot and subsequently the interaction with it when the robot is framed as undesirable competitor compared to when it is framed as a desirable assistant. The following is hypothesized:

**H3**: When a robot is described as competitor, a high level of interaction skills leads to a more negative evaluation of a) the robot’s sociability and b) its competence, which consequently leads to c) a more negative general evaluation of the interaction with the robot.

### Users’ individual background

In addition to the robot’s behavior and what future role people expect this robot to have, the individual background of the person interacting with the robot should have a great influence on how the robot and the interaction with it are evaluated. First of all, people’s technological background should be taken into account. On the one hand, there is technical affinity, which describes a person's positive attitude, excitement, and trust toward technology [19]. A high technical affinity resembles a high enthusiasm about new technologies [19], which should also lead to a very positive attitude towards robots in general. On the other hand, there is locus of control when using technology, which describes whether a person feels capable and confident or rather helpless and overwhelmed when handling technological devices [20, 36]. How competent a person feels handling technological devices usually relates to his or her actual technological competence and knowledge [20]. Thus, a high technological locus of control should lead to a more realistic view on what social robots are capable of, as it appeared to be the case in previous research [11].

Moreover, a person’s experiences with real robots but also with robots portrayed in mass media (real and fictional ones) should be considered. Most people have never interacted with a real robot before and thus a heavily used source of information for this still rather unfamiliar technology are media reports about real robots and, even more prevalent, science fiction representations (cf. [11]). Particularly since these portrayals often lack accuracy and elicit a biased picture of robots, their influences need to be considered.

Since negative expectations and fears regarding robots are often discussed in human-robot interaction research (cf. [9, 28, 37, 38]), as well as in literature (cf. [27, 33–35]), their influence should be taken into account as well. Especially the fear of robots gaining consciousness and becoming more intelligent and thus superior to humans represents a common science fiction scenario [30]. Since science fiction is widely spread and easily accessible for everyone, they are believed to have a great impact on people’s attitude towards social robots (e.g., [10, 11, 30]). Thus, the influence of people’s negative expectancies regarding social robots to become superior to and dominate humans is also considered in this study.

In conclusion the following hypotheses are postulated:

**H4**: In addition to the main and interaction effects of the robot’s level of interaction skills and the robot’s expected future role, people’s technological background, their previous experiences with robots, and their negative expectancies regarding robots influence people’s evaluation of a) the robot’s sociability and b) its competence, which consequently affects c) the general evaluation of the interaction with the robot.

## Material and methods

The laboratory study employed an experimental 2 (assistant vs. competitor expectation) x 2 (high vs. low level of interaction skills) between-subjects design. Participants were randomly assigned to the conditions. The ethics committee of the division of Computer Science and Applied Cognitive Sciences at the Faculty of Engineering of the University of Duisburg-Essen approved the study and written informed consent was obtained. The individual pictured in this manuscript has given written informed consent (as outlined in PLOS consent form) to publish these case details.

### Sample

Results of an a priori power analysis using G*power 3.1 software (based on 95% power and a medium effect size of f^2^ = 0.15; [39, 40]) recommended a sample size of 111 participants. Furthermore, our goal was to have at least 40 participants in each condition, resulting in a minimum size of 160 participants. In the end, 189 people participated in the lab study, of which 25 had to be excluded because they did not pass the manipulation check making sure people read the vignettes with sufficient attention (see next section). Additionally, one person was removed because of insufficient language skills and one because the vignette was accidently read after instead of before the interaction. Of the remaining 162 participants, 97 reported to be female and 65 to be male. The age ranged from 18 to 42 years with an average of 22.85 years (SD = 3.88). With regard to education, most participants were university students (94.4%) and either possessed university entrance-level qualifications (59.3%) or a university degree (38.9%).

### Experimental setting and procedure

First, a cover story was presented explaining that the participants are invited to the lab for a final evaluation of the robot’s improved interaction skills. After written consent was obtained from the participants, they were asked to read a vignette, where in the first part the robot Nao’s (SoftBanks Robotics) interaction skills were described as very elaborated. In the second part of the vignette, the robot was either described as competitor (Nao’s interaction and communication skills will be superior to human skills and it will take over many tasks which are currently executed by humans, because it will be able to do them in a more efficient, reliable, and safe way) or as assistant (Nao will be very helpful and assist humans with many exhausting and onerous tasks, to make them easier and more pleasant to do). This was followed by a manipulation check asking about the robot Nao’s interaction skills (possible answers: high, low, or medium) and its future role (possible answers: assistant, replacement, or no information on future application) as it was described in the vignette. Since only participants were included who passed this check, we are confident that we successfully manipulated people’s expectations with regard to the robot’s future role.

**Fig 1:**
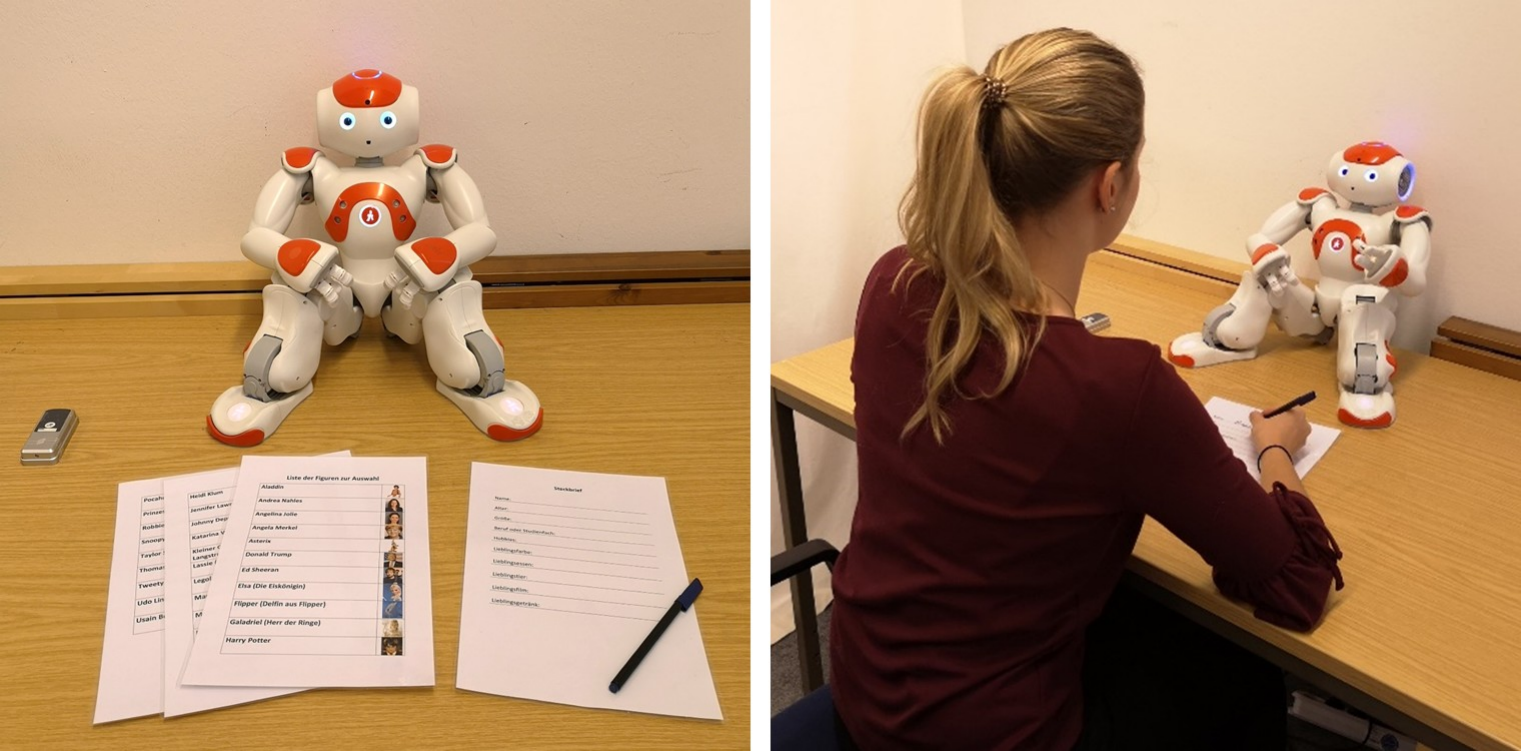
The experimental set-up.

For the subsequent interaction, participants sat in front of the robot Nao, which was placed in the middle of a table (see Fig 1). After the main functions of the robot were explained, the experimenter gave the robot a start signal and left the room. The robot then explained that it needed a moment for preparation and will notify the participant when it was ready to start. This gave the experimenter enough time to enter the adjacent room from which the robot was then controlled during the interaction. By using a webcam installed in the lab, the experimenter was able to see and hear the participant and to let the robot react in accordance with the participant’s answers (wizard of oz design; see [41]). To explain why the participants would be alone with the robot during the interaction, they were told that the robot produces a lot of data for the final evaluation which can only be processed by a high-performance computer located a few rooms down the hall. The experimenter explained that she wanted to make sure that the data will be transferred and saved correctly, which is why she would like to observe these processes during the interaction.

The webcam was justified by explaining that in case of error messages in the data, the developers of Nao’s interaction skills could use the videos to see what went wrong. Participants were also told that the robot would guide them through the interaction and that they should ring a bell when both interaction tasks were completed, which would notify the experimenter.

The first interaction task was a figure guessing game. Participants received a list with the names and small pictures of 32 different figures (celebrities as well as comic and movie characters) and were instructed to choose one of them without telling the robot which one they chose. The robot then asked different questions (e.g., “Is it a real person or a fictional figure from a comic or movie?”) to eventually guess the figure. A decision tree was constructed so that the robot was able to name the chosen figure after five questions. After three rounds of this game, the robot introduced the second interaction task: creating a profile about each other. Here, the robot and the participant took turns asking each other personal questions to fill out a given profile template (e.g., age, height, hobbies, and favorite movie).

In the high interaction skills condition, participants were able to communicate with the robot in a rather natural and sophisticated way. In the low interaction skills condition, the communication was more restricted (e.g., robot can only understand “Yes” or “No”), the robot deliberately applied some false grammar (e.g., false conjugation of verbs) and misunderstood the participants’ answers several times. After the two interaction tasks were completed, the experimenter came back to the lab and asked the participant to continue with questionnaires on the computer. At the end of the experiment, participants were debriefed and their time and effort were compensated with money or course credits.

### Questionnaires

#### Evaluation of the robot’s sociability and competence

To assess the robot’s perceived *sociability*, the Social Attractiveness subscale (Interpersonal Attraction Scale [42]; Likert scale ranging from 1 = “strongly disagree” to 5 = “strongly agree”; 5 items; e.g., “I think the robot Nao could be a friend of mine.”; α = 0.80) as well as the Sociability subscale (Source Credibility Scale [43]; semantic differential scale ranging from 1 to 5; 6 items; e.g., “unpleasant–pleasant”; α = 0.84) were adapted to ask about the robot Nao instead of a person. Additionally, the two subscales Perceived Enjoyment (5 items; e.g., “I enjoy the robot talking to me.”; α = 0.87) and Perceived Sociability (4 items; e.g., “I find the robot pleasant to interact with.”; α = 0.84) of the Acceptance of a Social Robot Scale ([44]; 1 = “strongly disagree” to 5 = “strongly agree”) were employed as well.

People’s evaluation of the *competence* of the robot Nao was assessed with the Task Attractiveness subscale (Interpersonal Attraction Scale [42]; Likert scale ranging from 1 = “strongly disagree” to 5 = “strongly agree”; 5 items; e.g., “The robot Nao would be a poor problem solver.”; α = 0.81) and the Competence subscale (Source Credibility Scale [43]; semantic differential scale ranging from 1 to 5; 6 items; e.g., “unintelligent–intelligent”; α = 0.89), after both scales’ items were adjusted to an interaction with a robot instead of with another person.

#### Evaluation of the interaction with the robot

To assess how people generally *evaluate the interaction* with the robot, adapted versions of the Evaluation subscale ([45]; 4 items; e.g., “I was enjoying the interaction with the robot Nao.”; 1 = „strongly disagree”to 5 = „strongly agree“; α = 0.81) and the Overall Rewardingness scale ([46]; 4 items; e.g., “The opportunity to interact with the robot Nao again is very desirable.”; 1 = „strongly disagree”to 5 = „strongly agree“; α = 0.85) were used.

#### People’s individual background

The participants’ *technological background* was measured with the Locus of Control when Using Technology Scale (KUT [20]; 8 items; e.g., “I feel so helpless regarding technical devices that I rather keep my hands off of them.”; 1 = “strongly disagree” to 5 = “strongly agree”; α = 0.85) and the Technical Affinity as Handling of and Attitude toward Electronic Devices Scale (TA-EG [19]; 19 items; e.g., “I enjoy trying an electronic device.”; 1 = “does not apply at all” to 5 = “applies completely”; α = 0.83).

Their *experiences with robots* were measured by asking participants how often (0 = “never”; 1 = “very rarely” to 5 = “very often”) they have had contact with real robots before. In a similar way, they were asked how often they have watched reports or something similar about real robots and how often they have watched fictional movies or series where robots played an important role before.

To assess participants’ *negative expectancies regarding robots*, 12 items asking about common science fiction scenarios were employed ([11]; e.g., “Robots will try to free themselves from humans.”; 1 = “strongly disagree” to 5 = “strongly agree”; α = 0.88).

#### Demographical background

Participants’ age, sex, educational level, and current employment or training status were assessed.

#### Further questionnaires assessed, but not analyzed for this paper

In addition, the Character subscale of the Source Credibility Scale [43], the Anxiety subscale of the Acceptance of a Social Robot Scale [44], Themes of Relational Communication: Trust, Dominance, and Equality [47], Contact Intentions [6], the General Anxiety subscale of the Frankenstein Syndrome Questionnaire [48] and Knowledge of Fictional Robot Characters [11] were assessed, but not used for the analyses of this paper.

## Results

For an overview of the descriptive values of the main influencing and dependent variables see Table 1.

**Table 1:** Descriptive statistics of the main influencing and dependent variables.

		Robot’s behavior				Robot’s expected future role					
		High skills		Lows skills		Assistant expectation		Competitor expectation		Total	
		*M*	*SD*	*M*	*SD*	*M*	*SD*	*M*	*SD*	*M*	*SD*
**Perceived competence**	Competence (SCS)	4.03	0.66	3.38	0.80	3.78	0.82	3.63	0.78	3.71	0.80
	Task Attractiveness (IAS)	4.11	0.61	3.53	0.72	3.86	0.68	3.78	0.78	3.82	0.73
**Perceived sociability**	Sociability (SCS)	4.32	0.53	3.97	0.65	4.23	0.55	4.06	0.67	4.14	0.61
	Social Attractiveness (IAS)	3.24	0.91	2.89	0.93	3.21	0.91	2.93	0.95	3.07	0.94
	Perceived Sociability (ASRS)	3.67	0.82	3.17	0.89	3.58	0.79	3.27	0.96	3.03	0.96
	Perceived Enjoyment (ASRS)	4.16	0.68	3.74	0.92	4.11	0.77	3.79	0.86	3.95	0.83
**Interaction evaluation**	Evaluation	4.23	0.58	3.61	0.87	4.08	0.72	3.77	0.85	3.92	0.80
	Overall Rewardingness	3.95	0.73	3.32	0.89	3.73	0.83	3.54	0.89	3.63	0.87

SCS = Source Credibility Scale [43]; IAS = Interpersonal Attraction Scale [42]; ASRS = Acceptance of a Social Robot Scale [44].

To test all hypotheses (H1 to H4), a structural equation model (SEM) was employed based on the theoretical deliberations outlined before. The process of structural equation modelling comprises several statistical techniques which enable analyses of the relationships between one or more, either continuous or discrete, independent variables and one or more, either continuous or discrete, dependent variables [49]. Structural equation modelling basically combines two multivariate techniques: multiple regression analysis and factor analysis [50]. On the one hand, it is possible to simultaneously estimate multiple dependence relationships akin to multiple regression equations and on the other hand, various measures can be incorporated for each concept similar to factor analysis [50]. A SEM has three major advantages over traditional multivariate techniques: measurement errors are explicitly assessed, latent (unobserved) variables can be estimated via manifest (observed) variables and a model can be tested to examine whether a conceptual or theoretical structure fits the data [51]. The aim of the current study is to examine the relative influences of a social robot’s interaction skills (H1), the robot’s expected future role (H2), a combination of the robot’s interaction skills and its expected future role (H3) and people’s individual background (H4) on a) the robot’s perceived competence and b) its perceived sociability and how these subsequently affect c) the general evaluation of the interaction with the robot.

**Fig 2:**
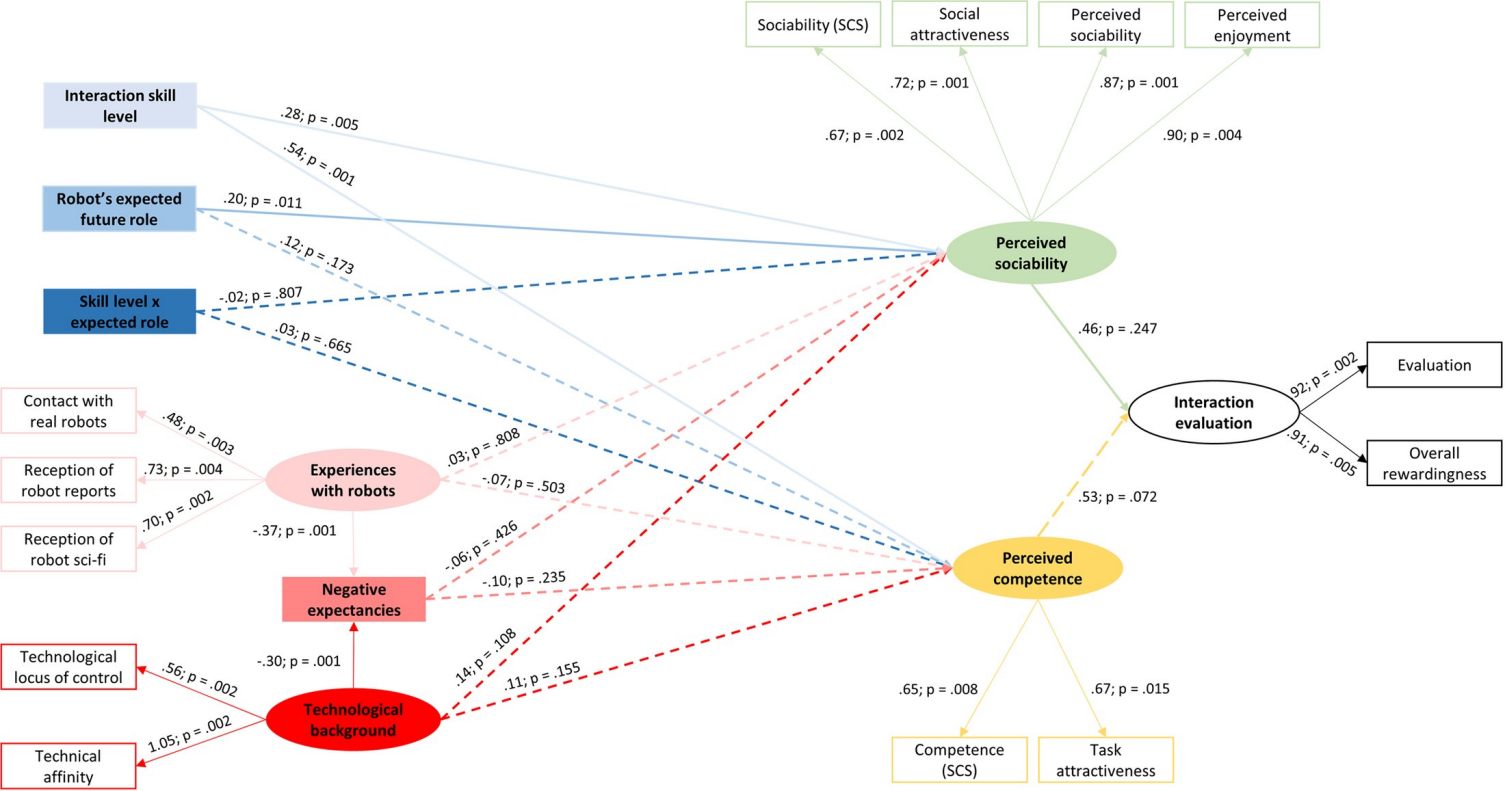
Structural equation model for the evaluation of the robot’s perceived competence and sociability as well as the general evaluation of the interaction.

For this SEM, *perceived sociability*, *perceived competence* and *interaction evaluation* as well as *technological background* and *experiences with robots* served as latent variables. Interaction skill level, expected future role, skill level x expected role, and negative expectancies regarding robots were included as manifest variables (see Fig 2).

The SEM analysis was computed using IBM SPSS AMOS 25.0 for Windows (IBM SPSS statistics, released 2017). The latent variable *perceived sociability* is made up of the four manifest variables sociability (Source Credibility Scale [43]), social attractiveness (Interpersonal Attraction Scale [42]), perceived enjoyment, and perceived sociability (Acceptance of a Social Robot Scale [44]). The latent variable *perceived competence* consists of the two manifest variables competence (Source Credibility Scale [43]) and task attractiveness (Interpersonal Attraction Scale [42]). The latent variable *interaction evaluation* consists of the two manifest variables evaluation and overall rewardingness. The latent variable *technological background* consists of the two manifest variables locus of control when using technology and technical affinity, while the latent variable *experiences with robots* consists of the three manifest variables contact with real robots, reception of reports about real robots, and reception of robot science fiction movies/series. The standardized model results for the latent dimensions can be found in Table 2.

**Table 2:** Coefficients of the manifest variables' loadings on the latent dimensions.

Latent dimension	Manifest variables	β	*SE*	*p*
**Perceived competence**	Competence (SC)	0.65	0.07	.008
	Task Attractiveness	0.67	0.06	.015
**Perceived sociability**	Sociability (SC)	0.67	0.05	.002
	Social Attractiveness	0.72	0.05	.001
	Perceived Sociability	0.87	0.02	.001
	Perceived Enjoyment	0.90	0.02	.004
**Interaction evaluation**	Evaluation	0.92	0.02	.002
	Overall Rewardingness	0.91	0.02	.005
**Technological background**	Technological Locus of Control	0.56	0.11	.002
	Technical Affinity	1.05	0.21	.002
**Experiences with robots**	Contact with real robots	0.48	0.09	.003
	Reception of robot reports	0.73	0.09	.004
	Reception of science fiction	0.70	0.08	.002

There were no missing data and variables were tested for multivariate normality and multicollinearity. For the evaluation of the model fit, standard criteria were applied following the suggestions from Hu and Bentler [52, 53]: The root mean square error of approximation (RMSEA; values below 0.08 indicate an acceptable fit), comparative fit indices (CFI/TLI; values above 0.90 indicate a good fit), and the standardized root mean square residual (SRMR; values below 0.08 indicate a good fit with the data). For this model, the RMSEA was 0.05, CFI was 0.96, TLI was 0.95, and the SRMR was 0.06, indicating an overall good model fit.

### Influence of the robot’s level of interaction skills

There was a significant direct effect of the robot’s interaction skill level on participants’ evaluation of the robot’s sociability (β = .54, *SE* = 0.08, *p* = .001) as well as competence (β = .28, *SE* = 0.08, *p* = .005), which confirms the assumptions of H1a and H1b. There was a significant indirect effect of the robot’s level of interaction skills on people’s general evaluation of the interaction with the robot via perceived sociability and competence (β = .41, *SE* = 0.07, *p* = .001), in support of H1c.

In summary, the robot’s behavior in terms of a high or low interaction skill level had a significant effect on participants’ evaluation of the robot’s sociability and competence, which also significantly affected people’s general evaluation of the interaction with the robot. The robot was evaluated as less sociable and less competent when it displayed a low level of interaction skills (see Table 1 for the descriptive values). Low interaction skills were also found to indirectly lead to a less positive general evaluation of the interaction with the robot (see Table 1).

### Influence of the robot’s expected future role

The expectation of the robot’s future role had a significant direct effect on the evaluation of the robot’s sociability (β = .20, *SE* = 0.08, *p* = .011), but not on the evaluation of its competence (β = .12, *SE* = 0.08, *p* = .173), which is in accordance with H2a but not with H2b. There was also only a marginally significant indirect effect of the expectation of the robot’s future role on the interaction evaluation via perceived sociability and competence (β = .15, *SE* = 0.07, *p* = .067). Therefore, H2c is not fully supported.

To sum up, how people evaluated the robot’s sociability was influenced by their expectation regarding the robot’s future role. When the robot was described to aim to become a threatening competitor in the future, this robot was evaluated less sociable than when it was described aiming to become a helpful assistant (see Table 1). The robot’s expected future role did not significantly affect how competent this robot was perceived. On a marginal significant level, people’s expectation regarding the robot’s future role indirectly affected their general evaluation of the interaction with the robot. When the robot was expected to become a competitor, the interaction with it was evaluated less positive (see Table 1).

### Combined influence of the robot’s level of interaction skills and its expected future role

There was no significant direct effect of the interaction variable (robot’s skill level x robot’s expected future role) on the robot’s perceived sociability (β = .03, *SE* = 0.08, *p* = .665) or competence (β = -.02, *SE* = 0.08, *p* = .807), which leads to a rejection of H3a as well as H3b. With regard to the combined influence of the robot’s expected future role and its displayed level of interaction skills, there was also no significant indirect effect on the general evaluation of the interaction (β = .00, *SE* = 0.08, *p* = .980), which leads to the rejection of H3c.

Summing up, there was no significant direct interaction effect of the robot’s level of interaction skills and the robot’s expected future role on the robot’s perceived sociability and its competence, as well as no significant indirect interaction effect on the general evaluation of the interaction with the robot.

### Influence of people’s individual background

With regard to individual backgrounds, participants’ technological background (β = -.30, *SE* = 0.12, *p* = .001) as well as their experiences with robots (β = .37, *SE* = 0.13, *p* = .001) had a significant direct effect on negative expectancies regarding robots. However, neither negative expectancies, nor technological background, nor experiences with robots had a significant direct effect on the evaluation of the robot’s sociability (negative expectancies: β = -.06, *SE* = 0.08, *p* = .426; technological background: β = .14, *SE* = 0.10, *p* = .108; experiences with robots: β = .03, *SE* = 0.13, *p* = .808) or competence (negative expectancies: β = -.10, *SE* = 0.09, *p* = .235; technological background: β = .11, *SE* = 0.11, *p* = .155; experiences with robots: β = -.07, *SE* = 0.14, *p* = .503). Likewise, there were also no indirect effects (via negative expectancies) of technological background and experiences with robots, neither on the robot’s perceived sociability (technological background: β = .02, *SE* = 0.03, *p* = .292; experiences with robots: β = -.02, *SE* = 0.04, *p* = .304) nor on its perceived competence (technological background: β = .03, *SE* = 0.04, *p* = .141; experiences with robots: β = -.04, *SE* = 0.04, *p* = .153). This leads to the rejection of the hypotheses H4a and H4b.

With regard to people’s evaluation of the interaction, experiences with robots (β = -.05, *SE* = 0.11, *p* = .615) as well as negative expectancies (β = -.08, *SE* = 0.08, *p* = .324) had no significant indirect effect on the general evaluation of the interaction. There was a marginally significant indirect effect of people’s technological background on the general evaluation of the interaction with the robot (β = .15, *SE* = 0.09, *p* = .053). Therefore, H4c is not fully supported.

In summary, none of the personality variables technological background, experiences with robots, or negative expectancies regarding robots had a significant direct effect on the evaluation of the robot’s sociability or its competence. There was also no significant indirect effect of people’s experiences with robots and their negative expectancies regarding robots on the general evaluation of the interaction with the robot. However, there was a marginally significant indirect effect of people’s technological background revealing that people with a higher affinity and competence with regard to technological devices appear to indirectly evaluate the interaction with the robot more positively.

### Post-hoc statistical power analysis

Post hoc power analyses using G*Power 3.1 were computed with the actual sample size of 162 participants. The statistical power for this study was 0.24 for detecting a small effect (f^2^ = .02) and exceeded 0.99 for a medium effect (f^2^ = .15) as well as for a large effect size (f^2^ = .35; Cohen, 1988). Thus, more than adequate statistical power was reached for medium to large effect sizes, but less than adequate power for a small effect size.

## Discussion

Since social robots are increasingly entering various areas of people’s daily lives, it is crucial to examine what affects people’s perceptions and evaluations of these robots and their interactions with them. Ultimately this will affect people’s acceptance of social robots and their willingness to use this technology [15]. For this study, our goal was to examine what influences people’s evaluations of specific attributes of a robot with which they just interacted, but also what subsequently influences people’s more general evaluation of the interaction with the robot. For this purpose we decided to look at three aspects which we assumed to have substantial influence: 1) the robot’s displayed level of interactions skills as a behavioral component, 2) what kind of role this robot is expected to have in the future (helpful assistant or threatening competitor), and 3) people’s individual background with regard to technology, experiences with robots, and negative expectancies regarding robots. After setting people’s expectation regarding the robot Nao to either become a threatening competitor or a helpful assistant in the future, they interacted with Nao, which then either displayed a low or high interaction skill level. That people had a personal encounter with a real robot in this study extends insights of studies which are based on surveys only and allows a more realistic situation to examine people’s evaluation of a robot’s characteristics and the interaction with this robot.

### The robot’s level of interaction skills

With regard to the robot’s behavior during the interaction, Rickenberg and Reeves [13] were among the first to emphasize the strong effects that the behavior of a non-human interaction partner has on people. They further explain that these effects “are not unilaterally good or bad; they can be either or both” and that “an animated character turns up the volume on social presence, which means that it can accentuate the effects of everything presented” (p. 55). In other words, the behavior of an interaction partner is of crucial importance when it comes to the perception and evaluation of this interaction partner. In accordance with these findings, the results of this study show that when a robot presents low interaction skills during people’s interaction with it, the robot is evaluated as less competent and less sociable compared to when the robot displays high interaction skills. Furthermore, these negative evaluations lead people to also generally evaluate the interaction with the robot less positively. The robot with low interaction skills was probably harder and less comfortable to interact with, which was accentuated by its displayed behavior during the interaction. Another considerable explanation is that this robot was also perceived as less useful, since a social robot displaying low interaction skills defeats its purpose of socially interacting with people [14]. And since perceived usefulness is known to influence the attitude towards as well as the usage intention of a technology [15], this could explain why the competence and the sociability of the robot displaying low interaction skills are evaluated more negatively. These negative evaluations of the robot’s attributes then cause people to also negatively evaluate the interaction with the robot in general.

Not many people have interacted with a social robot before and thus often rely on available information sources like mass media, which usually present a rather positively biased view on the skills of social robots [11, 30]. This was shown to lead to heightened expectations [11], which increases the possibility that these expectations are negatively violated when people interact with a real social robot which does not measure up to those high expectations [24, 26]. These violations of expectancies are linked to detrimental communication outcomes in interpersonal settings [32] and were also shown to lead to disappointment, mistrust, and rejection in human-robot interactions [24, 26]. This might have also happened in this setting since people were told that they will be interacting with a social robot in possession of sophisticated interaction skills. In case of the robot which then displayed low interaction skills, this could have caused a negative expectancy violation leading to a negative evaluation of this robot and the interaction with it.

### The robot’s expected future role

In addition to the display of different levels of interaction skills, it was examined what effects different expectations of the robot’s future purpose have. The robot which was portrayed as trying to compete with and eventually replace humans was evaluated as less sociable compared to the robot which was expected to become a beneficial assistant. Moreover, when people expected the robot to become a competitor, the negative evaluations of the robot’s attributes also lead them to generally evaluate the interaction with the robot more negatively–at least on a marginally significant level.

This confirms and extends previous research on the two different prominent views on social robots [11, 28, 31] by showing that framing a robot as competitor or assistant significantly influences how sociable this robot is perceived and also partly how an interaction with this robot is evaluated subsequently. A previous study showed that people who know more “bad” fictional robot characters have stronger negative expectancies regarding robots becoming the superior life form which will outrace and dominate us humans one day [11]. The current study extends these survey-based findings in a systematic-experimental way and shows that the fear of a robot becoming a threatening competitor also causes people to evaluate the sociability of the robot they just interacted with more negatively.

These findings hold important implications for the future shaping of the perception of social robots. If people have no access to personal experiences or existing categories into which something or someone can be encoded, they have to rely on statements and judgements from third parties [54]. Since social robots are not very common yet, many people base their knowledge about this technology on what they learn from others, a main source being mass media [11]. Here, the negative and fearsome view of robots developing their own agenda, revolting against and dominating humans is often promoted [28–30]. However, as this study shows, people’s reliance on third party information can also be used to shape people’s expectations and their subsequent evaluations regarding social robots in a positive way. This could be achieved by explaining and emphasizing the positive features and anticipated usefulness of social robots.

What kind of role people expected the robot to have in the future had no effect on how competent the robot was perceived, probably because in both descriptions the robot was described as having high interaction skills. After that, only the actual competence of the social robot, represented by its displayed level of interaction skills, seemed to be able to influence people’s evaluation of the robot’s competence. Also, the indirect influence of the robot’s expected future role on the general evaluation of the robot was rather weak. This can be explained by the circumstance that the robot’s perceived competence had a stronger influence on the interaction evaluation than its perceived sociability. The robot’s expected future role mainly influenced its perceived sociability and not so much its perceived competence.

### The robot’s expected future role influencing the perception of its interaction skill level

There was no direct interaction effect of the robot’s interaction skill level and the expectation of its future role on the evaluation of the robot’s competence or sociability. Consequently, there was also no indirect interaction effect on the general evaluation of the interaction with the robot. The expectation of the robot to become an assistant or a competitor seems not to have affected how the robot’s level of interaction skills were perceived. An explanation could be that the effect of the robot’s behavior during the interaction was too strong by itself to be influenced by what kind of role people expect this robot to have one day in the future. This is also supported by the effect sizes, which were largest for the robot’s level of interaction skills. The behavior is what people experienced live during their interaction with the robot, while the expected role was probably perceived as being far in the future and thus not as present as the behavior displayed live and in that moment. This explanation also goes in line with the insights by Reeves and Rickenberg [13], who emphasize the extraordinary influence of an agent’s behavior during an interaction on the subsequent evaluation of the agent.

Also, the low interaction skill level was probably brought to the fore during the interaction since people usually expect precision, sophisticated abilities, and high efficiency from robots [21, 23]. Since all these aspects were not given in the condition with the poorly skilled social robot, this robot was likely perceived as useless given its purpose of socially interacting with its environment [14]. This perception appears not to be affected by which role the robot is supposed to take over in the future, probably because with these poor skills the robot seems unsuitable for any kind of social setting.

### People’s individual background

In this study, we additionally considered various personality variables. The influence of people’s technological background, their experiences with real and fictional robots, and their negative expectancies regarding robots were taken into account while examining the main effects of the robot’s level of interaction skills and the robot’s expected future role. However, there was no significant effect found caused by these variables. There was a marginally significant indirect effect of people’s technological background on the general evaluation of the interaction with the robot. People with a more pronounced technological background, which includes their technical affinity and their locus of control when using technology, evaluated the interaction with the robot better. This can likely be traced back to these people being more enthusiastic about technology in general, which was already found in a previous study to lead to a more positive evaluation of the technology and the interaction with it [7].

An explanation for the overall not significant effects of people’s individual backgrounds could be that the effects of the robot’s expected future role and, even more, the robot’s behavior during the interaction were so strong that people’s technological background, their previous experiences with robots as well as their negative expectancies regarding robots did not make any further notable difference in the evaluation of the robot and the interaction with it. This again is supported by the findings of human-agent interaction studies, such as the one by Rickenberg and Reeves [13], which suggest that the behavior of an agent plays a pivotal role for the effect this agent has on a potential user.

### Limitations and future research

The study has some limitations regarding the generalizability of the results since most participants were students and thus predominantly young and highly educated. In future studies, the setting should also be moved to the participants´ personal environments and instead of a one-time event, interactions with the robot should take place several times over a longer period of time to examine long-term effects. Moreover, the dependent variable measures were self-reported, an experiment with behavioral measurements could bring further insights, for example, with regard to the willingness to interact with this robot and robots in general. However, we would like to emphasize that the participants of this study had a real interaction with a robot, which extend insights of studies solely based on surveys and offers a crucial basis for a more realistic evaluation of the robot’s characteristics and the interaction with this robot. In future studies, other pre-existing or interactional characteristics should be considered along with other factors which influence the perception and evaluation of a robot and the interaction with a robot. For instance, the appearance of a robot was found to have a great influence on people’s perception of it [1–4] and might also affect how the robot’s present and future role are perceived. Thus, appearance should also be considered in future studies dealing with the question which variables may influence the evaluation of a social robot.

## Conclusion

The current study provides novel insights regarding the effects of a social robot’s level of interaction skills as a behavioral component, the robot’s expected future role, and people’s individual backgrounds on the evaluation of this robot’s sociability and competence and subsequently on the general evaluation of the interaction with this robot.

Since knowledge of “malevolent” fictional robots was found to be related to negative expectancies regarding real robots [11], this study systematically examined whether a robot which is expected to become a threatening competitor is evaluated differently after people had a real interaction with it. Results showed indeed that the robot presented as a future competitor is evaluated as less sociable.

However, the most central insight from this study confirms previous findings [13] in showing that the behavior of the robot in the actual interaction has the most pivotal influence on how the robot and the interaction with it are evaluated. With the largest effect sizes of this study, a social robot displaying low interaction skills leads to a more negative evaluation of the robot’s sociability and competence as well as subsequently of the interaction with the robot.

Summing up, whether people expect a robot to become a competitor to them in the future plays an important role in how this robot’s sociability is evaluated. However, the robot’s behavior in the actual interaction appears to be the key variable influencing people’s evaluations of the robot and the interaction with it in general. Thus, when people get to the point where they interact with a social robot themselves, the robot’s behavior will be more decisive for how this robot is perceived and evaluated than people’s negative expectations regarding the robot’s future role or prior attitudes and personality traits.

## Supporting information

S1 File Complete data set. (SAV)
